# A multicenter prospective study evaluating use of EmboCube™ Embolization Gelatin alone or in combination with other embolic agents to control bleeding

**DOI:** 10.1186/s42155-025-00571-w

**Published:** 2025-05-31

**Authors:** Olivier Pellerin, Julien Frandon, Glen Schlaphoff, Ross Copping, Carole Déan, Warren Clements

**Affiliations:** 1 Department of Vascular and Oncological Interventional Radiology, Université de Paris, PARCC, INSERM, F-75006 Paris Assistance Publique-Hôpitaux de Paris, Hôpital Européen Georges Pompidou, Paris, France; 2 Department of Medical Imaging, IPI Plateform, Nîmes University Hospital, 30039 Nîmes, France; 3 Department of Interventional Radiology, Liverpool Hospital, Sydney, NSW Australia; 4 Department of Radiology, Alfred Health, Melbourne, VIC Australia; 5 Department of Surgery, Monash University School of Translational Medicine, Melbourne, Australia; 6 National Trauma Research Institute, Melbourne, Australia

**Keywords:** Bleeding, EmboCube™ Embolization Gelatin, Embolization, Gelatin sponge, Hemorrhage

## Abstract

**Background:**

Embolization is a vital endovascular procedure that can be used to quickly achieve hemostasis in patients experiencing uncontrolled bleeding. This study was conducted to describe real-world outcomes following embolization with a pre-cut absorbable gelatin sponge to control bleeding.

**Methods:**

This prospective study was conducted across five hospitals in Australia and France. Inclusion criteria included adults ≥ 18 years who required embolization with EmboCube™ Embolization Gelatin for bleeding. Primary performance and safety endpoints were the proportion of patients that achieved clinical success (i.e., cessation of bleeding post-embolization, the absence of rebleeding at the treated site requiring reintervention within 24 h), and the absence of unanticipated serious adverse device effects within 24 h of the initial embolization, respectively. Secondary endpoints included technical success and serious device- and/or procedure-related adverse events 28 days post-initial embolization.

**Results:**

A total of 101 patients (54 males) were enrolled and treated. Sixty-six patients were treated with EmboCube only, 35 patients were treated with an additional embolic to control bleeding. Technical and clinical success rates were 100% and 99%, respectively. No patient experienced an unanticipated serious event related to the embolic. The mean time to hemostasis was 3.4 (± 3.96) minutes. Of the 90 patients that completed 28 days of follow-up, 4 (4.4%) experienced an adverse event (access site hematoma, *n* = 2; ischemic colitis, *n* = 1, peritonitis, *n* = 1).

**Conclusion:**

EmboCube is safe and effective for control of acute and sub-acute arterial bleeding, alone or in combination with other embolic agents, when rapid hemostasis is required.

**Trial registration:**

Clinicaltrials.gov. Registered 23 March 2022, https://clinicaltrials.gov/study/NCT05307783.

**Graphical Abstract:**

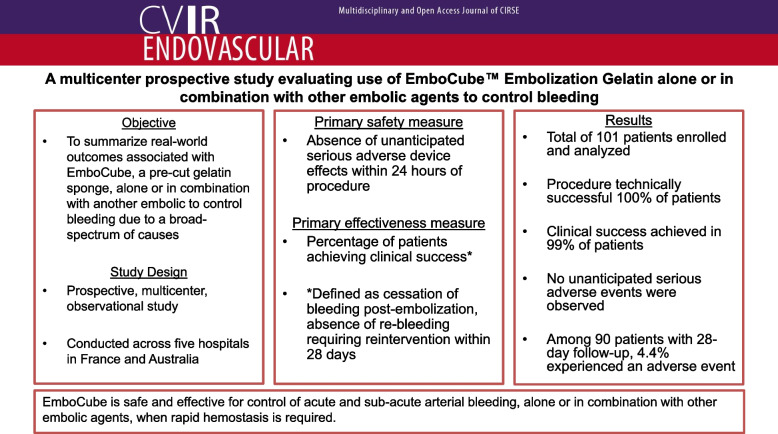

## Introduction

Bleeding can result from trauma- (e.g., pelvic, visceral) and non-trauma-related (e.g., spontaneous, gynecologic, iatrogenic) causes [1, 2]. The inability to control bleeding is a leading cause of preventable death [3]. Hemorrhage control is critical to lowering mortality [4]. Embolization is an expedient nonsurgical intervention often used to achieve hemostasis in diseased or ruptured vasculature [5–7]. Several types of temporary (e.g., absorbable gelatin sponge) and permanent (e.g., coils, calibrated microspheres, liquid agents such as N-Butyl-Cyanoacrylate or NBCA) embolics are available for the occlusion of blood vessels [5, 8–13]. The type of embolic, or combination of embolic agents, to control bleeding is determined based on several factors such as vessel size, location, flow dynamics, extent of hemorrhage, pre-existing coagulopathy, and physician preference [14, 15].

Of the available products, gelatin-based embolics have been used as an intravascular embolic agent for over 50 years [5, 16]. Since its first reported use in 1945 [17], several studies have reported successful use of gelatin-based embolics for cessation of trauma-related bleeding [9, 18] and to stop bleeding in areas such as the gastrointestinal tract, spleen, uterus/pelvis, and kidney [19–32]. In general, gelatin-based embolics (i.e., gelatin sponge) are considered a safe and effective agent to control bleeding. According to the Eastern Association for the Surgery of Trauma (EAST) guidelines, gelatin sponges are particularly recommended in locations where rapid but temporary occlusion may be desired, commonly following pelvic trauma when used non-selectively [33]. Although the clinical utility of gelatin-based embolics has been recognized by different treatment guidelines, there is no universal standard regarding which embolic, or combination of agents, to use [34, 35]. As a result, treatment practices can vary across indications.

In 2022, the gelatin-based embolic, EmboCube™ Embolization Gelatin, received the Conformité Européenne (CE) Mark in Europe. A beneficial aspect of EmboCube is its availability in the form of pre-cut cubes. Prior to the emergence of EmboCube, clinicians manually cut gelatin sheets, which was a potentially time-consuming task resulting in non-uniform particles. Evidence indicates that EmboCube is associated with high rates of technical and clinical success [36, 37]. However, there is an on-going need for data confirming its benefit to patients. This study describes clinical outcomes following use of EmboCube in real-world practice to control bleeding due to a broad spectrum of causes among patients in Australia and France.

## Methods

### Study design

This was a prospective study conducted across five centers, two in France, three in Australia. To be eligible for inclusion in the study, consecutive patients were required to be ≥ 18 years of age, require embolization for bleeding that could be performed in accordance with the device’s Instructions for Use, and provide written and informed consent. The full list of country-specific inclusion and exclusion criteria are provided in Table 1. The eligibility criteria were broad as a key objective of this study was to capture real-world use of EmboCube Embolization Gelatin (Merit Medical Systems, Inc., South Jordan, Utah, USA), including the wide spectrum of bleeding indications, treated vessels, and patient profiles.

**Table 1:** Country-specific inclusion and exclusion criteria

France	Australia
**Inclusion criteria**	
1. Age ≥ 18 years 2. Patient requires embolization and is suitable for treatment with EmboCube in accordance with the device’s Instructions for Use for the treatment of bleeding 3. Patient provides written informed consent to study data collection 4. Patient has Social Security	1. Age ≥ 18 years 2. Target vessel(s) require treatment are ≤ 5 mm 3. Patient requires embolization and is suitable for treatment with EmboCube in accordance with the device’s Instructions for Use for the treatment of bleeding 4. Patient provides written informed consent to study data collection
**Exclusion criteria**	
1. Patient has bleeding site in the neck, head, or brain 2. Patient has co-morbidity with survival prognosis of less than 30 days, in the opinion of the treating physician 3. In the investigator’s opinion, participation in the study may not be in the patient’s best interest 4. Patient is pregnant woman, under guardianship or curatorship, under judicial protection, and/or does not write or speak French	1. Vascular anatomy precludes correct catheter placement 2. Feeding arteries are too small to accept selected EmboCube Embolization Gelatin 3. Patient has bleeding site in the neck, head, or brain 4. Presence or suspicion of vasospasm 5. High-flow arteriovenous shunts with a diameter greater than the selected EmboCube Embolization Gelatin 6. Use in the pulmonary vasculature 7. Use in pre-operative portal vein embolization 8. Patient has severe atherosclerosis 9. Patient has known allergy to gelatin of porcine origin 10. Patient has co-morbidity with survival prognosis of less than 30 days, in the opinion of the treating physician 11. In the investigator’s opinion, participation in the study may not be in the patient’s best interest

This study was conducted in compliance with the Declaration of Helsinki, Good Clinical Practice, and independent ethics committees and local and national regulatory requirements. The study is reported according to the STROBE guidelines.

### Device

EmboCube is a sterile ready-to-use device that consists of biocompatible, hydrophilic, and dry pre-cut cubes of resorbable 100% porcine gelatin packaged in a 10-mL syringe with a standard luer lock tip. All available sizes (2.5 mm and 5.0 mm) and weight configurations (25 mg, 50 mg, 100 mg) of EmboCube were available for use in this study.

### Procedure

All procedures were performed in accordance with the standard operating protocols at each participating center and the device’s Instructions for Use. EmboCube sizes were selected after the diameter of the vessel(s) responsible for bleeding was measured. Following hydration of EmboCube within the 10 mL syringe using a 1:1 mixture of 0.9% saline and iodinated contrast (Omnipaque 300/350, GE Healthcare, Chicago, Illinois, USA), under fluoroscopic imaging, the embolic was injected into the delivery microcatheter positioned at the target vessel requiring occlusion. EmboCube was administered according to flow and hemorrhage dynamics, after waiting for a short period of time, typically 1- to 2-min intervals (determined by the treating physician), flow was observed before check angiography was performed to confirm vessel occlusion. All procedures were performed by certified interventional radiologists (OP, JF, GS, RC, WC) with > 20 years of experience in arterial embolization.

### Study measures and outcomes

The primary effectiveness endpoint was clinical success, defined as cessation of bleeding post-embolization and the absence of rebleeding at the treated site requiring reintervention (i.e., repeat embolization or additional surgery) within 24 h following the index procedure. The primary safety endpoint was the absence of unanticipated serious adverse device effects within 24 h following the index procedure.

Secondary endpoints included the following: (1) the incidence of adverse events (serious and device and/or procedure-related) to 24 h post procedure; (2) the incidence of adverse events (serious and device- and/or procedure-related) between 24 h and 28 days post procedure; (3) technical success, defined as successful occlusion of the target area determined by the physician based on post-embolization angiography; and (4) the incidence of device observations relating to EmboCube.

An independent physician adjudicator was responsible for systematically reviewing and adjudicating all serious adverse events related to the device and procedure, including deaths, as well as any adverse event potentially related to the device and procedure. The independent physician adjudicator was experienced in interventional peripheral vascular procedures to occlude blood flow and control bleeding and was entirely independent of the study sponsor and all participating centers.

### Analysis

Study measures and outcomes were summarized using descriptive statistics. Mean, standard deviation, and median were used to summarize continuous data; categorical data were reported using percentages. As this study was purely descriptive, no hypothesis testing was conducted.

## Results

### Patient demographics

A total of 101 patients were enrolled and treated, 51 were from France and 50 were from Australia. Over the 28-day study period, a total of 4 patients were lost to follow up and 7 patients died. Table 2 summarizes patient demographics and medical history. Approximately half of all patients were male (53.5%). The three most prevalent comorbidities reported in patients’ medical history were hypertension (40.6%), active malignancy (28.7%), and history of bleeding (24.8%). Fifty-three patients (52.5%) were on an anticoagulant/antiplatelet medication at the time of hemorrhage. The most common anticoagulant/antiplatelet medications were enoxaparin (24.8%) and aspirin (17.8%).

**Table 2:** Patient demographics, medical history, and baseline medications

Patient Demographics	Patients (*N* = 101)
Age (years), mean ± SD (N)	57.1 ± 20.37 (101)
Males, n/N (%)	54/101 (53.5)
*Race, n/N (%)*	
African American/Black	2/101 (2.0)
Asian	6/101 (5.9)
Caucasian/White	40/101 (39.6)
Pacific Islander	5/101 (5.0)
Not reported/Refuse to disclose	25/101 (24.8)
*Ethnicity, n/N (%)*	
Hispanic or Latino	4/101 (4.0)
Not Hispanic or Latino	72/101 (71.3)
Not Reported/Refuse to disclose	25/101 (24.7)
Weight (kg), mean ± SD (N)	75.3 ± 16.50 (92)
Body mass index, mean ± SD (N)	26.5 ± 5.22 (91)
*Medical History, n/N (%)*	
History of bleeding	25/101 (24.8)
History of alcohol abuse	10/101 (9.9)
History of active malignancy	29/101 (28.7)
History of Bleeding disorders^1^	10/101 (9.9)
Anemia	6/10 (60.0)
Aplastic anemia	1/10 (10.0)
Disseminated intravascular coagulation	1/10 (10.0)
Sickle cell anemia	1/10 (10.0)
Von Willebrand disease	1/10 (10.0)
Hemorrhagic shock	1/10 (10.0)
History of cerebrovascular disease	13/101 (12.9)
History of diabetes (Type 2)	18/101 (17.8)
History of cirrhosis of the liver	8/101 (7.9)
History of hepatic (liver) failure	8/101 (7.9)
History of gastrointestinal bleeding	9/101 (8.9)
History of hypertension	41/101 (40.6)
History of prior pregnancy complications	9/47 (19.1)
History of renal insufficiency/failure	9/101 (8.9)
* Baseline Anticoagulant, Antiplatelet Medication, n/N (%)*	
Antiplatelets	
Aspirin	18/101 (17.8)
Prasugrel (Effient)	1/101 (1.0)
Clopidogrel (Plavix)	1/101 (1.0)
Anticoagulants	
Apixaban (Eliquis)	10/101 (9.9)
Dabigatran (Pradaxa)	1/101 (1.0)
Enoxaparin (Lovenox)	25/101 (24.8)
Heparin	10/101 (9.9)
Rivaroxaban (Xarelto)	3/101 (2.9)
Warfarin (Coumadin)	2/101 (2.0)
Idarucizumab	1/101 (1.0)

^1^Patients can have more than one type of bleeding disorder

### Procedural characteristics

Table 3 summarizes procedural characteristics. The most common indication for embolization was trauma (32.7%). The mean procedure time was 66.9 min (± 43.74) and the mean time to hemostasis following embolization was 3.4 min (± 3.96). The main vessel used for access was the femoral artery (85.1%).

**Table 3:** Procedure Characteristics

Characteristic	Patients (*N* = 101)
*Indication for Index Procedure, n/N (%)*	
Trauma	33/101 (32.7)
Postpartum hemorrhage	13/101 (12.9)
Hypervascular tumors	23/101 (22.8)
Gastrointestinal system disorders	7/101 (6.9)
Angiodysplasia	1/7 (14.3)
Inflammation of the gastrointestinal tract	1/7 (14.3)
Other gastrointestinal complication^1^	5/7 (71.4)
Other Cause of Bleeding	25/101 (24.8)
Soft tissue hematoma	11/34 (32.4)
Post-operative bleeding	14/34 (41.2)
*Procedure Details*	
Procedure Time (minutes), mean ± SD (N)	66.9 ± 43.74 (101)
Total fluoroscopy time (minutes), mean ± SD (N)	22.6 ± 17.85 (100)
Total amount of contrast (mL), mean ± SD (N)	134.1 ± 92.73 (97)
Time to hemostasis post-EmboCube embolization (minutes), mean ± SD (N)	3.4 ± 3.96 (100)
*Procedure Access Location, n/N (%)*	
Femoral artery	86/101 (85.1)
Radial artery	13/101 (12.9)
Other^2^	2/101 (2.0)
*Patients’ treated vessels, n/N (%)*^*3*^	
Hepatic artery	15/112 (13.4)
Internal iliac artery branch	30/112 (26.8)
Other abdominal artery branch	6/112 (5.4)
Parietal artery	24/112 (21.4)
Renal artery	8/112 (7.1)
Splenic artery	3/112 (2.7)
Superior mesenteric artery	4/112 (3.6)
Uterine artery	22/112 (19.6)
* Maximum Reference Vessel Diameter (mm), mean* ± *SD (N)*	3.3 ± 1.43 (84)

1 Cecal bleed on apixaban (*n* = 1), duodenal ulcer (*n* = 1), gastric ulcer (*n* = 2), ulcer (*n* = 1)

2 Left ulnar artery (*n* = 1), humeral artery (*n* = 1)

3 Patients could have more than one treated vessel

The mean total volume of prepared EmboCube administered was 13.8 mL (± 17.55). A total of 171 devices were used in this study; the median number of devices was 1.0 per patient. The most commonly used sizes were 5.0 mm, 50 mg (55.6%, 95/171) and 2.5 mm, 50 mg (28.1%, 48/171).

Sixty-six patients (65.3%) were treated with EmboCube only. Thirty-five patients (34.7%) were treated with additional embolic agents. Table 4 summarizes the specific embolic agents used.

**Table 4:** Embolic devices used during the study

Embolic Agents	Patients (*N* = 101)
Patients treated with EmboCube only	66/101 (65.3%)
Patients treated with EmboCube and other embolics^1^	35/101 (34.7%)
Coils^2,3^	33/35 (94.3%)
Non-coil permanent agent	
Liquid agents	
Onyx	2/35 (5.7%)
Squid-18	2/35 (5.7%)
Non-coil permanent agents	
Permanent embolics	1/35 (2.9%)
Embosphere®	3/35 (8.6%)
Other unspecified non-coil embolic	6/35 (17.1%)
Resorbable agent	
Other gelatin embolic	1/35 (2.9%)

1 Patients could be treated with different embolic agents

2 One patient was treated with both coils and onyx

3 One patient had both coils and Embosphere®

Technical success was achieved for all treated patients. No evidence of unintended embolization was observed during the index procedure.

### Effectiveness and safety outcomes

Clinical success was achieved in 100 of 101 treated patients (99%). No difference in clinical success was observed among patients treated with EmboCube only compared to EmboCube in combination with another embolic agent.

One patient required a re-intervention within the 24-h period following the index procedure to treat bleeding that occurred within the vessels treated as well as several other vessels that subsequently required embolization. For this patient, the indication for the index procedure was a biopsy tract-related bleed in the right lumbar L3 artery with prophylactic embolization of the adjacent lumbar arteries (L1, L2, L4). Although the treated area was successfully occluded in this patient, due to evidence of deterioration on the same day of the procedure, a repeat angiogram was performed, and embolization of the same vessels was deemed necessary using coils and NBCA. The adverse event reported for this patient was attributed to therapeutic anticoagulant usage which could not be ceased. No patient experienced an unanticipated serious adverse event related to EmboCube within 24 h of the procedure.

Among the 90 patients that completed the 28-day follow up, 4 patients (4.4%) experienced a serious adverse event between 24 h and 28 days following the procedure. These four adverse events are summarized in Table 5.

**Table 5:** Summary of adjudicated serious adverse events due to EmboCube or the embolization procedure

Adverse event	Days to onset of event	Site-reported outcome
Hematoma ≥ 5 cm at puncture site, with or without surgical repair	2	Resolved with sequelae
Hematoma of the left kidney	6	Resolved/Recovered
Ischemic Colitis	5	Resolved/Recovered
Peritonitis	2	Ongoing

A total of 7 patients died during the study. None of the deaths were related to EmboCube, or the embolization procedure. Causes of death were cancer (*n* = 1) 16 days post procedure, cirrhosis (*n* = 1) 23 days post procedure, multi-organ failure (*n* = 1) 6 days post procedure, and progressive deterioration of liver function (*n* = 1) 11 days post procedure. Two patients died due to unspecified sequelae of blood loss, one 11 days after the index procedure and another 23 days post procedure.

## Discussion

This study describes clinical outcomes following use of a pre-cut absorbable gelatin sponge to control bleeding in patients treated at hospitals in Australia and France. Over one third of the patients included in this study underwent embolization due to trauma (32.7%). The high rates of technical success (100%) and clinical success (99%) suggest that the pre-cut gelatin sponge used can effectively contribute to the delivery of life-saving care to critically unwell patients across a variety of indications alone, or in combination with other agents. Among the patients who died during the study period, none of the deaths were device- or procedure-related. As there were no unanticipated serious adverse events associated with the embolic used, and the rate of all-cause serious adverse events were low (4.4%), its safety profile is considered tolerable, especially within the context of the urgent care required.

The favorable performance and safety profiles of EmboCube reported in this study align with results from prior studies that have documented the clinical utility of gelatin-based embolics for a variety of bleeding indications [25–31, 38]. For example, a study published in 2021 describing clinical outcomes in 23 patients treated with Gelfoam® in combination with coils for hemorrhoidal bleeding reported a technical success rate of 100% [38]. In that study, clinical success, defined as a sustained ≥ 2-point improvement in the French bleeding severity score for ≥ 6 months with no complications, was achieved in 20 of the 23 patients (87%) [38]. A study published in 2024 that retrospectively analyzed 50 patients with pelvic arterial bleeding who were treated with Gelfoam between January 2020 and May 2021 reported a technical success rate of 100% and no complications [31]. In that study, the all-cause mortality rate was 10%, however none of the deaths were related to the device or the procedure.

Currently, there is no universal standard regarding which embolic agent is preferred for different bleeding indications; the choice of embolic agent(s) remains the discretion of the treating physician. Any embolic agent should be suited to the individual anatomy, flow dynamics, and urgency of a patient’s situation. A benefit of the EmboCube product is its pre-cut delivery in a sterile syringe, which allows rapid rehydration and administration, a particular advantage for patients actively bleeding. This is a tangible and time-saving solution compared to the need for the operator to manually cut and load traditional gelatin sponge into solution, which is commonly available in a sheet format designed for topical use. This is an important advantage as the standardization of EmboCube allows uniform pledgets to be injected each time leading to more predictable outcomes. In addition, the EmboCube platform is faster to prepare than many other embolic agents including NBCA, squid, onyx, and detachable coils.

In this study, physicians were allowed to treat patients with more than one embolic agent, based on their clinical judgement. As a result, this study provides valuable insight regarding real-world patient outcomes from care under different physicians from different practices. An important strength of this study is that it expands the current body of literature by providing evidence to suggest that there is no detrimental impact of periprocedural use of other embolic agents with a resorbable gelatin embolic. An additional strength of the present study is that it contained a larger sample of patients with diverse indications and different vessels requiring intervention, relative to prior studies focused on outcomes in smaller cohorts of patients with a single indication for the embolization procedure.

A key aspect of the literature to date is not the novel and minimal invasive nature of a gelatin-based agent in embolization procedures, but rather its ability to provide clinicians an essential and potentially life-saving resource for patients with urgent need [31]. This is of particular importance in Australia where endovascular use of the gelatin-based embolic Gelfoam is not currently approved for commercial use. Based on the high rates of technical and clinical success, as well as the low incidence of adverse events reported in this study, we anticipate that this study will assist healthcare stakeholders, including regulatory bodies, recognize the ability of a pre-cut gelatin-based embolic, alone or in combination with other agents, to meet the needs of urgent care for patients with bleeding.

This study was designed to capture clinical outcomes in real-world practice; all participating sites were requested to enroll consecutive patients to minimize selection bias. Nevertheless, this study was subject to certain limitations. As a purely descriptive uncontrolled cohort study, no hypothesis testing was conducted. Additionally, the time to hemostasis was not standardized across centers. While this study describes clinical outcomes of patients treated with EmboCube, 35 patients (34.7%) were treated with an additional embolic(s), although there was not a significant difference in clinical success. It is unclear whether use of additional embolics represented failure of EmboCube or standard practice patterns at each hospital. Use of a pre-cut gelatin-based embolic to control bleeding may necessitate use of additional embolics based on physician judgement of the vessel(s) to be treated. Despite these limitations, the present study does provide useful information regarding the safety and performance of EmboCube across different indications and vessels requiring intervention to control bleeding in France and Australia. The high rates of technical and clinical success, with few incidences of adverse events, give credence to the safety and effectiveness of the device used in this study, and may encourage its use more broadly for patients who require life-saving care to control bleeding, irrespective of whether other embolic agents were used previously, periprocedurally, or after EmboCube as part of standard practice.

## Conclusions

Findings from this real-world study regarding the use of EmboCube, a pre-cut absorbable gelatin sponge, to treat bleeding in patients from France and Australia demonstrated that this embolic is associated with few adverse events and high rates of clinical and technical success, alone or in combination with other agents. As there is no consensus regarding which embolic agent should be used to control bleeding, findings from this study suggest that as a cost-effective, time-proven gelatin sponge, EmboCube should be considered as part of the embolization armamentarium wherever or whenever embolization procedures are required.

## Data Availability

Data are not publicly available to comply with patient confidentiality.

## Abbreviations

CE: Conformité Européenne
EAST: Eastern Association for the Surgery of Trauma
NBCA: N-Butyl-Cyanoacrylate
